# Eight years tumor control with pazopanib for a metastatic resistant epithelioid hemangioendothelioma

**DOI:** 10.1186/s13569-014-0018-3

**Published:** 2015-04-23

**Authors:** Olivia Bally, Louis Tassy, Bertrand Richioud, Anne-Valérie Decouvelaere, Jean-Yves Blay, Olfa Derbel

**Affiliations:** Sarcoma Unit, Centre Léon Bérard, 28, Laennec street, 69008 Lyon, France

**Keywords:** Epitheloid hamangioendothelioma, Pazopanib, Liver

## Abstract

Epithelioid hemangioendothelioma is a rare connective tissue tumor of vascular origin. It is most commonly found in young to middle aged women, and its clinical behavior is remakably variable from an indolent metastatic tumor to an aggressive rapidly growing neoplasm. Most tumors are diagnosed in an advanced unresectable phase and when clinically aggressive, require systemic cytotoxic treatment of sarcoma. Then, the 5-year survival rate after chemotherapy does not exceed 30%. Antiangiogenics are active in selected sarcoma subtypes: pazopanib, the only anti angiogenic registered agent for sarcoma provides a median PFS of 4.5 months only in the pivotal study. Their activity in EHE has been reported but long term outcome of these patients remain unreported. We report a case of a female patient with HEH who was treated with pazopanib for almost 8 years. Pazopanib therapy resulted in clinical improvement of symptoms and durable stabilization of liver tumors and lung lesions.

**Conclusion:** Pazopanib is a promising therapeutic option in patients with HEH.

Epithelioid hemangioendothelioma (EHE) is a rare vascular tumour first reported as intravascular bronchioloalveolar tumor by Dail and Liebow (1975) in a patient with a lung variant of the disease (LEH) [1]. EH has been also named sclerosing interstitial vascular sarcoma and sclerosing endothelial tumor, before the term EH was introduced by Weiss and Enzinger [2] as a clinical state between benign hemangioma and angiosarcoma. EHE originate from endothelial cells and generally occurs in the liver, lungs, and other soft tissues. Positivity of endothelial differentiation markers, such as factor VIII-dependent antigen, CD-34, and CD-31, as well as vascular invasion and an infiltrating growth pattern (except in the portal region) are important in the diagnosis of epithelioid hepatic hemangioendothelioma [3,4]. Specific mutation involving the 1p36.3 chromosomal region been demonstrate in subsets of these tumors [5]. Chronic liver disease is not considered a significant cause of HEHE [6] .In almost half of the cases, HEHE is diagnosed at metastatic stage mostly with pulmonary and bone localization [7]. EH is characterized by an often unpredictable clinical course. Whereas some patients present a rapidly progressive disease, others may remain stable for several years. However, overall, HEHE have a poor prognosis: 5-year overall survival of HEH patients after standard primary radical treatment is 30% and therefore, it is one of the liver malignancies associated with a better prognosis [8]. There are few standard options and consensus reports for the management of EHE. Surgery with partial hepatectomy or liver transplantation is the first-line treatment when HEH is confined to the liver. Chemotherapeutic agents reported as treatment regimens are Doxorubicin, Vincristine, Fluorouracil (5-FU), and Interferon (IF)-alpha 2b [3]. Indolent EHE stable and non evolutive without any treatment have also been reported. Vascular endothelial growth factor (VEGF) and the VEGF receptor are detectable on EHE tumor cells [9], suggesting VEGF role in EHE growth. VEGF inhibitors may have therapeutic value: a phase II study reported partial response to bevacizumab in EHE patients [10]. Sangro reported a case of a young male patient with EHE who was treated with sorafenib for almost 2 years [11].

Pazopanib has recently been approved by the U.S. Food and Drug Administration (FDA) and by the European Medicines Agency (EMA) for the treatment of advanced renal cancer and soft-tissue sarcomas (STS). We present here first report of clinical activity of pazopanib in EHE with a still ongoing very long term progression free survival.

## Case presentation

**Figure 1:**
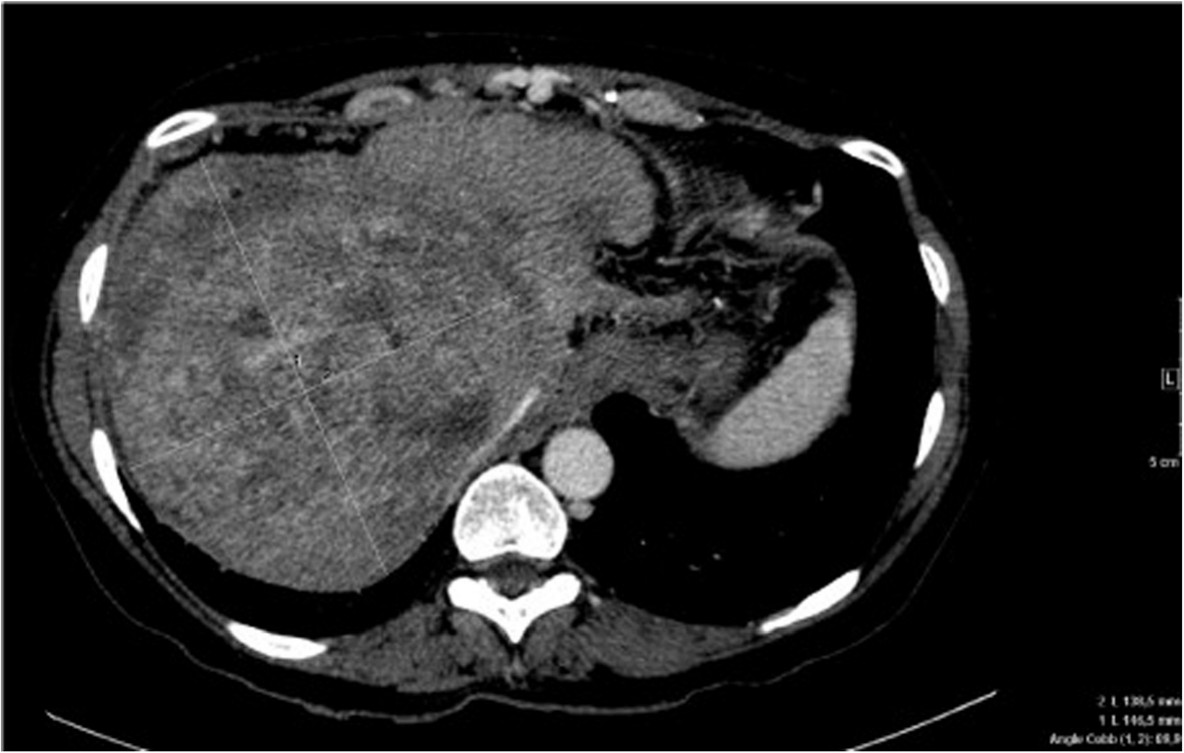
Computing tomography scan showing multifocal abnormalities, involving both lobes of the liver. The uninvolved portions of hepatic parenchyma underwent relative hypertrophy. No calcification was visible initially. In the left liver, lesions coalesce, forming a diffuse pattern. So the target lesion was chosen in the right liver, initially measuring 138 × 146 mm, extending to the capsular margin with areas of retraction. After administration of IV contrast material, this main nodule displayed marginal enhancement during the portal phase. Inferior vena cava was compressed but remained permeable.

A 38 years old female patient, with unremarkable medical history, presented an acute abdominal pain suggesting a cholecystis. Patient had a cholecystectomy and per operatory, a liver nodule was discovered and biopsied. The results showed a fibrotic tissue containing poligonal tumor cells with round nuclei and a relatively scarce cytoplasm containing vacuolae with sinusoidal growth pattern. Immunohistochemically, tumor cells had a positive staining by CD-34, (cytoplasmatic diffuse 100%), CD-31 (cytoplasmatic diffuse 100%) and was negative to CK7 and CK20. The final pathologic diagnosis was hepatic epitheliod hemangioendothelioma. Then, patient was followed without any treatment. In March 2001, patient was recovered in our center, referring right shoulder pain. A chest and abdominal computed tomography (CT) scan showed multiple bilateral pulmonary nodules and multiples hypodense liver lesions suggestive of metastases. Surgery was not advised, as disease was extended to lung and hepatic lesions were bilobar. Patient started systemic treatment with Doxorubicin 75 mg/m2, every 3 weeks. CT scan evaluation after three months reported stable disease. Treatment was continued until six cycles of chemotherapy with stability, and then patient was followed every 3 month. Ten months after, CT scan showed hepatic progression. Multidisciplinary sarcoma committee suggested including patient in the 62011 study. Patients received Brostacilline for eight cycles with stability of hepatic lesions for duration of 21 months [12]. Shortly after, the symptoms dramatically worsened with abdominal pain and digestive haemorrhage episode related to a portal hypertension due to the hepatic invasion. The patient was then proposed to participate to a clinical trial testing the GW786034 antiangiogenic agent within the study 62043 of EORTC [13]. Treatment was started, in December 2005 at 800 mg daily. Shortly afterwards, patient developed grade 3 hepatic toxicity, dose was further reduced to 400 mg allowing transaminase normalisation. After 12 weeks of treatment, a significant improvement of symptoms was noticed, analgesic requirement was reduced and hepatomegaly disappeared. CT scan assessment after 3 months demonstrated stable hepatic and pulmonary disease (Figure 1). Side effects reported were grade 2 anaemia and fatigue. The patient remains on 400 mg of pazopanib without significant toxicities and with ongoing clinical and radiological benefit during than 3 years. Liver transplantation was discussed but not considered due to the presence of lung metastasis. Three years after the beginning of pazopanib, treatment discontinuation was again required because of grade 2 hepatic toxicity. As the abdominal pain increased, whereas hepatic biology improved, patient was rechallenged pazopanib treatment at 200 mg daily with a progressive increase to 400 mg, based on a biological monitoring twice a week. Clinical benefit was rapidly noticed with particularly abdominal pain control, allowing an improvement in patient’s quality of life.

**Figure 2:**
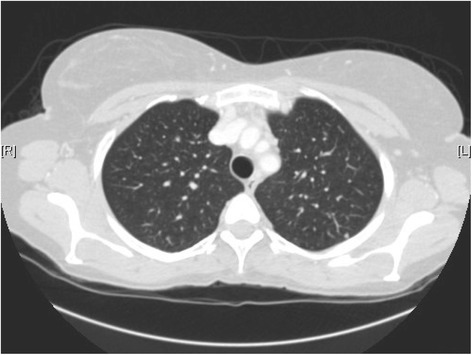
Chest scanner of November 2007 showing micronodular pulmonary lesions.

**Figure 3:**
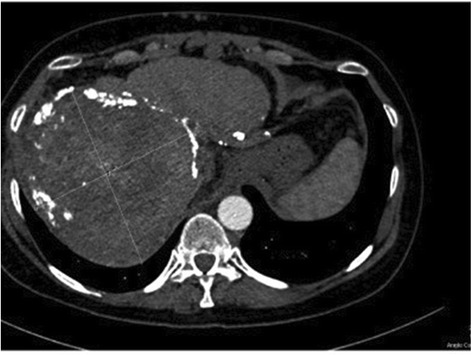
CT scanner of April 2014 showing that the appearance and size of the main lesion remained similar. The target lesion measured 126*137 mm and peripheric calcifications had appeared confirming radiological tumoral control.

**Figure 4:**
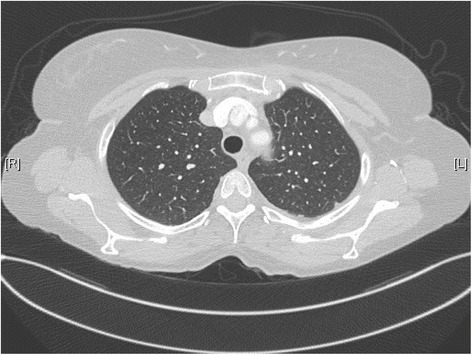
Chest scanner of October 2014 showing stable aspect of lung micronuodules.

Patient is still on 400 mg of pazopanib, PS 0, for over 100 months, with stability of the pulmonary nodules, a minor tumor size reduction observed in the liver and progressive calcification and tumor shrinkage. Altogether, using Response Evaluation Criteria In Solid Tumors criteria v. 1.0,10 [14], tumor response consisted in a prolonged stabilization (Figures 2, 3 and 4). Importantly an ectasia of the thoracic was noted on the most recent echocardiography, this anomaly will be carefully monitored. The role of pazopanib in this phenomenon observed after 8 years of treatment remains unclear but should prompt further investigations in long term survivors under VEGFR2 inhibitors including pazopanib.

## Discussion

The present case report describes a typical case of HEHE. The symptoms (abdominal pain, hepatomegaly), the site of metastases (lung) and the bilobar liver involvement are all characteristic of the initial presentation of this tumor. This report demonstrates for the first time that pazopanib is an active treatment, providing a very long disease control in a patient in third line treatment of a progressive multimetastatic epithelioid hemangioendothelioma (HEHE). HEHE was first described by Ishak et al. [15] as a group of rare borderline vascular tumors with primary liver involvement characterized by the presence of epithelioid endothelial cells. Although many patients (25–42%) are asymptomatic at the time of diagnosis [7], the most frequent symptoms include pain, weight loss, fatigue, and jaundice [3,7]. Imaging studies have an important role in the diagnosis of HEHE. Tumor lesions appear as solid, non- homogenously hypodense nodules with a ring-like, low-density border, and a lower-density centre in contrast-enhanced CT imaging. Also, magnetic resonance imaging (MRI) shows non-homogenously hypointense lesions on T1-weighted images and hypertense lesions on T2-weighted images (Lin). In this case, the tumoral lesions were observed as hypodense. The diagnosis of HEHE must be confirmed by an expert pathologist examination of the tissue. Histopathological characteristics include an invasive growth pattern with preservation of the liver acinar composition and portal tracts. The tumour consists of epithelioid or dendritic cells, which are characterized by large eosinophilic cytoplasm in a fibromyxoid stroma [16]. In the current case, both typical tumour architecture and immunohistochemical positivity of endothelial cell markers (such as CD-31 and CD-34) supported the diagnosis of HEHE.

A small number of single case reports of HEH patients treated with cytotoxic chemotherapy have been published. Patients with EHE still alive 5 years after initial treatment with a doxorubicin-containing regimen, i.e. long-term survivors do not exceed 8% [17]. The most frequently used agent is doxorubicin [18], or its liposomal form [19]. However, the results have been disappointing and HEH is considered a poorly chemosensitive tumor. VEGF expression’s observed in hepatic EHE specimens increases interest in employing anti- angiogenic therapy for this disease. Lenalidomide, sorafenib and sunitinib were tested in HE treatment and reported to be efficient in recent cases reports [20-23], and a phase 2 study [24].

Pazopanib, a synthetic indazolyl pyrimidine, is a novel multitargeted TKI interfering with several tumor environment factors that play a key role in a broad spectrum of tumor types. Pazopanib is a second-generation small-molecule TKI with high affinity against VEGFR-1/2/3 and with a lower affinity against PDGFR-α/β, FGFR-1/2 and stem cell factor receptor (c-KitR) [25].

The response achieved in our case was a prolonged stabilization associated with significant intratumoral changes. This long progression free survival is interpreted to be due to pazopanib effect, because of previous progression, clinical improvement. In fact, pulmonary nodules remains stable, whereas minor tumor size reduction was observed in the liver, with progressive calcification and tumor shrinkage. Calcification and other changes in tumor density without clear tumor shrinkage are considered as highly suggestive of tumor response, especially after the introduction of the newest antineoplastic molecular-targeted therapies [26]. Overall, pazopanib was well tolerated and the relative lack of side effects compared to standard chemotherapy allows this exceptional duration of treatment, reported for the first time. The observation of aortic ectasia requires further investigation.

In conclusion, pazopanib demonstrated a long stabilization of HEHE, with dramatic improvement in clinical status. We think that this treatment is a promising therapeutic option in patients with HEH. Further research and prospective studies are required to contribute to the data regarding the natural history and pazopanib effectiveness in this rare tumor.

Pazopanib is the first antiangiogenic drug that has shown successful results in a phase III trial in STS [20]. It was well tolerated in advanced STS and demonstrated interesting antitumor activity in pretreated patients with leiomyosarcomas, synovial sarcomas, and other eligible STS entities.

## Consent

Written informed consent was obtained from the patient for publication of this Case Report and any accompanying images. Copy of the written consents is available for review by the Editor-in-Chief of this journal.
